# Age effect on in vitro fertilization pregnancy mediated by anti-Mullerian hormone (AMH) and modified by follicle stimulating hormone (FSH)

**DOI:** 10.1186/s12884-020-02875-2

**Published:** 2020-04-09

**Authors:** Han-Chih Hsieh, Jia-Ying Su, Shunping Wang, Yen-Tsung Huang

**Affiliations:** 1 Institute of Statistical Science, Academia Sinica, 128 Academia Road, Taipei, 11529 Taiwan; 2 Women and Infants Hospital in Rhode Island, 101 Dudley St, Providence, 02905 RI USA

**Keywords:** Anti-Mullerian hormone, Follicle stimulating hormone, In vitro fertilization, Mediation analysis

## Abstract

**Background:**

Both follicle stimulating hormone (FSH) and anti-mullerian hormone (AMH) are widely used to assess the ovarian reserve in women for in vitro fertilization (IVF). However, studies also showed that both AMH and FSH are significantly associated with age: as age increases, AMH decreases and FSH increases. This study aims to investigate the mechanism of age effect on IVF live birth rate, particularly through mediation and interaction by AMH and FSH.

**Methods:**

We conducted a retrospective cohort study of 13970 IVF cycles collected by eIVF from 2010 to 2016. A series of logistic mixed models were used to estimate the association of live birth and AMH (or FSH). The mediation effects and proportion mediated, were quantified by our previously proposed mediation analyses. We further investigated the FSH-modified mediation effects on live birth rate through AMH, accounting for the nonlinear age effect.

**Results:**

Our analyses showed that age effect on live birth was mediated more by AMH than by FSH (18 vs. 6%). The mediation effect through AMH can be further modified by FSH level regardless of women’s age.

**Conclusions:**

In summary, mediation model provides a new perspective elucidating the mechanism of IVF successful rate by age. The majority of the age effect on live birth rate remained unexplained by AMH and FSH, suggesting its importance and independent role in IVF.

## Background

For female infertility patients, follicle stimulating hormone (FSH) and anti-müllerian hormone (AMH) represent the two most frequently utilized ovarian reserve tests that serve as important markers reflecting successful in vitro fertilization (IVF) outcomes. FSH is a member of the glycoprotein hormone family, produced by the gonadotropic cells of the anterior pituitary gland [1]; AMH is a dimeric glycoprotein hormone exclusively produced by granulosa cells [2–4]. Previous work suggested that low AMH confers to a lower likelihood of live birth, while low FSH confers to a higher likelihood of live birth [5–7]. Yet, it is suggested that AMH >0.8-1.0 ng/ml is indicative of normal ovarian reserve, while FSH >10 mIU/ml is indicative of diminished ovarian reserve [6]. In addition to the dose-response relationship between AMH (or FSH) and the probability of live birth, both markers are also found to be significantly associated with age: as age increases, AMH decreases and FSH increases [8–10]. Furthermore, numerous studies have identified that as women’s age increases, the success live birth rate of IVF decreases [11–13].

Based on the associations mentioned above, this study aims to further investigate the mechanism of age effect on IVF live birth rate, particularly through mediation by AMH and FSH. Mediation serves as a mechanism linking a risk factor (e.g., age) and an outcome (e.g., live birth rate). Mediation analysis was first proposed in psychology literature where mediation effect was identified by a product of two associations: one between the risk factor and the mediator and the other between the mediator and the outcome conditional on the risk factor [14, 15]. For the past two decades, mediation analysis has been extensively developed under the framework of causal inference [16, 17]. Using the notions of counterfactual outcome [18], causal definitions and their identifiability assumptions have been carefully studied [19, 20]. In addition to providing rigorous theoretical foundation, causal mediation analysis enables us to conduct mediation analyses for the setting where the mediator and the outcome are non-continuous with possible exposure-by-mediator nonlinear interactions [21–23]. We framed our scientific question by a mediation model where we had a dichotomous AMH or FSH (high vs. low) and a dichotomous live birth outcome (yes vs. no), and the effects of interest were the direct effect (DE) of age on the risk of live birth and the indirect effect (IE) mediated through AMH or FSH.

The main purposes of this study were therefore to quantify the mediation effects of age on live birth through AMH or FSH, incorporating the interactions between age and both intermediary factors. Additionally, we examined whether the age effect on live birth mediated through AMH is further mediated by FSH level.

## Methods

### Patient selection

A retrospective cohort analysis was conducted using the electronic medical record database eIVF, designed by PracticeHwy.com (Dallas, Texas). The dataset we obtained consisted of 144,044 cycles extracted from 60 different IVF centers in the United States from 2000 to 2016. Information recorded in the eIVF database for each cycle included a distinctive numeric patient identifier, patient’s age at cycle start, the date of the oocyte retrieval, cycle status, cycle outcome, AMH, FSH, estradiol, along with other 80 variables. Our work involved the de-identified eIVF dataset and was determined to be exempt by Institutional Review Board of Women and Infants Hospital of Rhode Island.

**Fig. 1:**
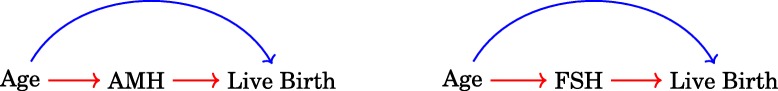
Causal diagrams with age as the exposure, AMH or FSH as the mediator, and live birth as the outcome

Since the assessment of AMH had not been widely adopted in clinical use until the year of 2010, cycles before 2010 were excluded resulting from the missing AMH values. Cycles with missing or incomplete information were all excluded, as well as those were outliers in certain variables, such as age, cycle number, total number of oocytes retrieved, number of 2PNs, number of follicles, the value of E2, AMH and FSH. Centers that provided with less than 10 cycles were considered unreliable, thus were removed. Therefore, the final dataset contained 13790 autologous IVF cycles, with known AMH, FSH values and confirmed information about live birth or not. Details about the inclusion and exclusion criteria of the study were documented as a flowchart in Fig. 1 in the previous paper [24] and in our Supplementary Materials.

### Statistical methods

To observe the marginal effects of age, AMH and FSH, a series of logistic mixed effect models were used to investigate the association of live birth and AMH (or FSH), adjusted for potential confounders: age, body mass index (BMI) and random effects of centers, as well as involving the interactions between age, AMH and FSH. Random effects of centers were included to account for the heterogeneity across numerous centers.

Figure 1 shows the two causal diagrams that depict the direct (blue) and indirect (red) effects in a mediation model. One with AMH, the other with FSH as the mediator; both with age as the exposure and live birth as the outcome. In order to fit the single-mediator model we developed based on the methods estimating mediation effect of both dichotomous mediator and outcome [23], we converted the original continuous AMH and FSH values into dichotomous values to reflect their clinical significance: AMH less than 1.0 ng/ml and FSH less than 10 mIU/ml were considered as 0s; FSH greater than or equal to 10 mIU/ml and AMH values greater than or equal to 1.0 ng/ml were considered as 1s. We then calculated the risk ratios (RR) and confidence intervals (CI) of the direct effects (DE) and indirect effects (IE) through AMH and FSH with and without adjusting the interaction with age, by using the bootstrap method and the mediation models we proposed. We reported direct and indirect effects on the scale of risk ratios (RR), comparing the live birth rate of women with age of 39 (the 3rd quantile) vs 32 (the 1st quantile).

Additional mediation analysis was conducted with cycles grouped based on the cut-off values we derived from using the quintiles of FSH (Q1=5.1, Q2=6.5, Q3=7.7, Q4=9.7 mIU/ml). Mediation models were applied to each groups by FSH level to identify the direct and indirect effects of age on live birth in relation to AMH. Furthermore, in order to take the nonlinear effect of age into consideration, cycles were also grouped based on patients’ age: all ages were rounded to the nearest integer, while patients <30 years of age were considered as 30, and patients >40 years of age were considered as 40. By sorting the cycles based on their FSH and age, we then obtained a 5-by-11 matrix, each unit within the matrix contained cycles with FSH and age under certain cutoffs. The first group out of the 55, with the least FSH (<5.1 mIU/ml) and age (<30 years) value, was defined as the reference group. Mediation analysis was performed on the rest 54 groups, comparing to the reference, to determine the direct effects, indirect effects, and proportion mediated. The proportion mediated was defined as the proportion of indirect effect in the total effects; more specifically, log risk ratios of indirect effect divided by the sum of log risk ratios of direct effect and log risk ratios of indirect effect.

## Results

The demographic characteristics and live birth rate among patients of age 30, 35, 37, and 40 years old can be found in Table 1. The average age (year ± standard deviation (SD)) of the patient population is 35.0 ±4.7. FSH significantly increased from 6.5 mIU/ml to 8.8 mIU/ml as age increased from <30 to >40 years old (*p*<0.001), while AMH decreased significantly from 4.0 ng/ml to 1.2 ng/ml (*p*<0.001). The live birth rate had a statistically significant decrease of 28.9% as the ages increased from <30 to >40 years old (35.9% vs 7.0%, *p*<0.001).

**Table 1:** Demographic characteristics and live birth rate of all five different age groups

	All	Age					
		<30	30-35	35-37	37-40	>40	
N	13790	2202	4739	1913	2763	2173	
	Mean (SD)	Mean (SD)	Mean (SD)	Mean (SD)	Mean (SD)	Mean (SD)	*p*-values
BMI (kg/ *m*^2^)	25.9 (6.0)	26.0 (6.2)	25.7 (6.0)	26.1 (5.9)	26.1 (5.9)	26.1 (5.8)	0.012
Estradiol (pg/ml)	2261 (1485)	2732 (1673)	2436 (1508)	2277 (1483)	1950 (1274)	1751 (1220)	<0.001
FSH (mIU/ml)	7.6 (3.8)	6.5 (2.9)	7.2 (3.3)	7.6 (3.9)	8.2 (4.1)	8.8 (4.6)	<0.001
AMH (ng/ml)	2.4 (2.7)	4.0 (3.4)	2.9 (2.9)	2.1 (2.3)	1.5 (1.7)	1.2 (1.3)	<0.001
Live Birth (%)	23.5	35.9	29.9	23.5	15.4	7.0	<0.001

As shown in Table 2, logistic regression models for live birth, AMH and FSH were performed using age, AMH, FSH, adjusting the confounding factors and exposure-mediator interactions. Simple regression models were performed to assess the marginal effects of age, AMH and FSH, showing that all three were notably associated with live birth (*p*<0.001). As expected, the dose-response relationship between AMH and live birth was positive, and the dose-response relationship between FSH and live birth was negative. To explore whether including the exposure (age)-mediator (AMH or FSH) interaction terms might improve the models, several more regressions were performed that supported significant age-by-AMH (or FSH) interactive effect on live birth (*p*<0.05). We also performed regressions that included both AMH and FSH as well as their interaction terms with age. While the interaction between FSH and age was significantly associated with live birth, the interaction between AMH and age was not. Additional logistic regression models, shown in Table 3, were perform to estimate the marginal effects of age and FSH on AMH, adjusting the same confounding factors. Similarily, effects of age and AMH on FSH were also estimated. Both mediators turned out to be significantly associated with one another. (*p*<0.001)

**Table 2:** Odds ratio estimates on live birth as the outcome

Live Birth	Model	Age	AMH	FSH	AMH*Age	FSH*Age
Marginal Effect	M1	0.89 (0.88, 0.90)	-	-	-	-
	M2	-	2.32 (2.11, 2.54)	-	-	-
	M3	-	-	0.55 (0.49, 0.62)	-	-
Individual Effect	M4	0.90 (0.89, 0.91)	1.68 (1.52, 1.86)	-	-	-
	M5	0.89 (0.88, 0.90)	-	0.69 (0.61, 0.78)	-	-
	M6	0.88 (0.87, 0.90)	0.71 (0.33, 1.55)	-	1.03 (1.00, 1.05)	-
	M7	0.89 (0.89, 0.90)	-	2.63 (0.97, 7.10)	-	0.96 (0.94, 0.99)
Joint Effect	M8	0.90 (0.89, 0.91)	1.61 (1.45, 1.78)	0.80 (0.71, 0.91)	-	-
	M9	0.89 (0.88, 0.91)	0.87 (0.39, 1.96)	2.45 (0.87, 6.89)	1.02 (1.00, 1.04)	0.97 (0.94, 1.00)

M1-M3 are logistic regression models for live birth assessing the marginal effects of age, AMH and FSH adjusting bmi and random effect of centers.

M4-M7 are logistic regression models for live birth assessing the individual effects of AMH and FSH adjusting age, bmi, random effect of centers, and considering the interactions between age, AMH and FSH.

M8-M9 are logistic regression models for live birth assessing the joint effects of AMH and FSH adjusting age, bmi, random effect of centers, and considering the interactions between age, AMH and FSH.

For M1-M9, their respective variances of the random effects estimates are 0.47,0.41,0.45,0.44,0.46,0.46,0.44,0.43,0.44

**Table 3:** Odds ratio estimates on AMH and FSH as the outcomes

		Model	Age	AMH	FSH
AMH	Marginal Effect	M1	0.84 (0.84, 0.85)	-	-
		M2	-	-	0.18 (0.16, 0.20)
	Individual Effect	M3	0.85 (0.85, 0.86)	-	0.21 (0.19, 0.23)
FSH	Marginal Effect	M4	1.11 (1.10, 1.12)	-	-
		M5	-	0.18 (0.16, 0.20)	-
	Individual Effect	M6	1.06 (1.04, 1.07)	0.21 (0.19, 0.23)	-

M1-M2 are logistic regression models for AMH assessing the marginal effects of age and FSH, adjusting bmi and random effect of centers.

M3 is a logistic regression model for AMH assessing the individual effect of FSH adjusting age, bmi, random effect of centers.

M4-M5 are logistic regression models for FSH assessing the marginal effects of age and AMH, adjusting bmi and random effect of centers.

M6 is a logistic regression model for FSH assessing the individual effect of AMH adjusting age, bmi, random effect of centers.

For M1-M6, their respective variances of the random effects estimates are 0.49,0.62,0.53,0.26,0.28,0.27

A single-mediator model was used to assess the effects of both AMH and FSH on the probability of live birth. With the Q1 and Q3 of exposure we derived earlier, the results were observed in Table 4 that comparing patients who were 39 years old to those 32 years old, the effect mediated through AMH accounted 16% (95%CI: 14, 18%) of the total effect by age, the effect mediated through FSH accounted for only 4% (95%CI: 4, 5%). Additional mediation analysis conducted by involving the exposure-mediator interactions revealed similar results (18% vs 6%).

**Table 4:** Risk ratio estimates of direct and indirect effects of age on live birth

	Direct Effect	Indirect Effect		Proportion Mediated	
	Risk Ratio*	*p*-values	Risk Ratio*	*p*-values	Proportion
AMH	0.57 (0.54, 0.59)	<0.001	0.90 (0.89, 0.90)	<0.001	0.16 (0.14, 0.18)
FSH	0.53 (0.50, 0.56)	<0.001	0.97 (0.96, 0.97)	<0.001	0.05 (0.04, 0.06)
AMH + int	0.57 (0.54, 0.60)	<0.001	0.88 (0.87, 0.90)	<0.001	0.18 (0.16, 0.21)
FSH + int	0.53 (0.50, 0.56)	<0.001	0.96 (0.95, 0.97)	<0.001	0.06 (0.05, 0.08)

∗Effects were estimated for the comparison of live birth rate between women with 39 years old and 32 years old.

Table 5 demonstrates the direct effect (DE), indirect effect (IE), and proportion mediated obtained from applying our mediation model to cycles grouped based on the quintiles of FSH. Since AMH played a more important role in mediation as shown in Table 4, here we considered AMH as the mediator, and FSH as the effect modifier. Results showed that comparing patients with an average of 39 years old to those with an average of 32 years old, the mediation effect through AMH accounted for 20% (95%CI: 14,26%) among patients with the least FSH value (<5.1mIU/ml, *n*=2720), which decreased monotonically to 7% (95%CI: 2, 11%) for patients with the highest FSH value (>9.7mIU/ml, *n*=2766). The result suggested that AMH mediated the age effect on live birth (7%-20%), which was modified by FSH level.

**Table 5:** Risk ratio estimates of direct and indirect effects of age on live birth with AMH as the mediator for different FSH groups

FSH (N)	Direct Effect		Indirect Effect		Proportion Mediated
	Risk Ratio*	*p*-values	Risk Ratio*	*p*-values	Proportion
<5.1 (2720)	0.63 (0.55, 0.69)	<0.001	0.89 (0.87, 0.91)	<0.001	0.20 (0.14, 0.26)
5.1 ∼6.5 (2743)	0.60 (0.53, 0.67)	<0.001	0.92 (0.90, 0.94)	<0.001	0.14 (0.10, 0.20)
6.5 ∼7.7 (2699)	0.65 (0.58, 0.72)	<0.001	0.93 (0.91, 0.95)	<0.001	0.14 (0.09, 0.20)
7.7 ∼9.7 (2862)	0.61 (0.54, 0.67)	<0.001	0.94 (0.93, 0.96)	<0.001	0.11 (0.07, 0.15)
>9.7 (2766)	0.45 (0.38, 0.51)	<0.001	0.95 (0.92, 0.98)	0.003	0.06 (0.02, 0.11)

∗Effects were estimated for the comparison of live birth rate between women with 39 years old and 32 years old.

**Fig. 2:**
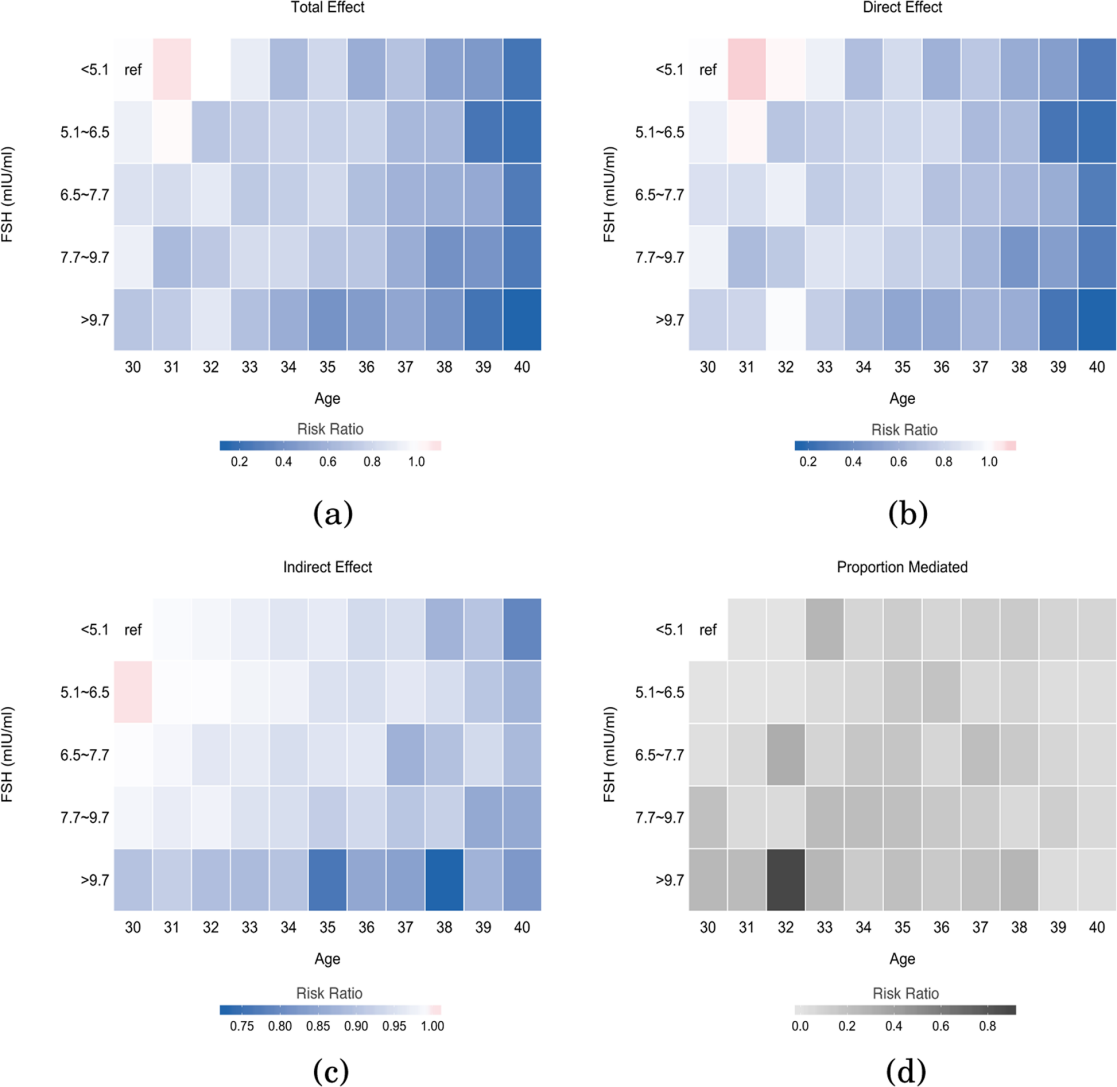
Estimated **a** total effect, **b** direct effect, **c** indirect effect and **d** proportion mediated with AMH as the mediator for patients from different age groups combined with different FSH groups

An additional analysis was conducted in order to further explore the nonlinear effect of age. As shown in Fig. 2, where total effect (TE), direct effect (DE), indirect effect (IE) and proportion mediated are presented in heatmaps. The increase of direct effect among different age and FSH groups was mostly dominated by the increase of age (Fig. 2b); whereas the increase of indirect effect among different age and FSH groups was mostly dominated by the increase of FSH (Fig. 2c). However, all heatmaps indicated the trend that the total effect (TE), direct effect (DE), indirect effect (IE) increase as the FSH and age increase. Therefore, the heatmaps provided a comprehensive visualization of the path-specific effects between age, AMH and live birth, modifying age and FSH values. The analysis incorporating the nonlinear age effect further supported the result in Table 5. The indirect effect of age increased mediated through AMH significantly decreased the live birth rate, and such mediation effects were notably modified by FSH level regardless of women’s age.

## Discussion

The principal finding of this study was that the age effect on live birth was more strongly mediated by AMH and less by FSH. By comparing patients who were 39 years old to those 32 years old, 16% of live birth resulted from a specific pathway: age affected AMH, which then in return affected live birth; FSH, on the other hand, accounted for only 4% of the age effect on live birth, indicating that FSH is a less significant mediator in the effect of age on live birth. However, aside from the statistically significant mediation effects through AMH and FSH, the direct effect of age still accounted for the majority of live births, which suggests that age affected live birth through pathways other than through AMH or FSH.

The etiology of age affecting live birth rates through pathways other than AMH or FSH may be multi-factorial. Currently available diagnostic tests that are heavily influenced by women’s age, such as estradiol, inhibin B, the antral follicle count (AFC), the ovarian volume (OVVOL) and the ovarian blood flow, are also measures to predict the ovarian reserve and chance of live birth besides AMH and FSH [6]. Moreover, the reduction of live birth rates is not linked solely to the reduction of number of oocytes, but also to the reduction in the number of embryos and implantation rate, which studies have also found to be affected by age [11]. The biology underlying the association is potentially related to the impaired decidual and placental development and embryo interaction with the uterus [25]. In addition, medical disorders that are developed more commonly in older women, such as high blood pressure or diabetes, can lead to higher risk of fatal death, thus decrease the live birth rates [26].

Of note, since the interaction effects between AMH (or FSH) and age were statistically significant as shown in Table 2, the interaction terms were thus added in the mediation models to accommodate the relationships, yielding a more positive finding. The interaction effects indicate that even with the same levels of AMH or FSH, the resulting live births of every cycles under patients with different age may still vary. According to the estimates of the random effect of centers in both Tables 2 and 3, there exists variation for the effect of different centers across the United States on the live birth rate, and the mixed model effectively accounts for the variability of center effects. The age-varying effects may suggest that markers of ovarian aging or uterus aging other than AMH or FSH affecting the probability of live birth contribute to the effects, such as inhibin B, estradiol, antral follicle count(AFC) and uterine factors [27]. More studies are needed to provide additional evidence for the roles of these biomarkers determining the interaction effects between age and AMH (or FSH).

Through categorizing FSH and applying the mediation analyses, we were able to identify that the proportion mediated by AMH on live birth decreased as FSH value increased (Table 5). This finding suggests that the effects of AMH on live birth may vary depending on FSH level and that the increase of FSH would decrease the mediation effect of AMH. However, this approach, as well as the previous analysis, underlay the assumption of the linear relationship between age and live birth. The nonlinear effect between age and live birth has been well-documented by previous studies [28–30]. To account for this, the analysis presented in Fig. 2 was conducted using heatmaps, providing more sophisticated results on interpreting the mediation effects of AMH. Interestingly, although the direct effect (DE) of age, decreased the live birth rate as age increased; whereas the indirect effect (IE) of age through AMH decreased the live birth rate as FSH increased. The interaction between FSH and AMH presenting in the indirect effect (IE) of age on live birth rate through AMH (Fig. 2c), was supported by early studies where both markers were found regulating the development of ovary and follicular growth at different phases. While the granulosa cells only develop the FSH receptors during the development of secondary follicles, AMH expression starts in the granulosa cells of early primary follicles, inhibiting the recruitment of primordial follicles [31, 32].

Our study has several limitations. Since patients with poor prognosis were more likely to undergo multiple cycles before they achieve live birth, they may contribute a larger proportion of failed cycles in the dataset. This may in return underestimate the mediation effects of AMH (or FSH), and the likelihood of live birth. In addition, although the center-level heterogeneity was adjusted by including the random effects for each center in all models that we applied to characterize the association of live birth and AMH (or FSH), we were still unable to account for the differences in AMH (or FSH) assay due to the diversity of geographic areas encompassed and centers queried.

## Conclusion

In conclusion, the results of this study suggest that the age effect on live birth was mediated more by AMH than by FSH, and that the mediation effect through AMH can be further modified by FSH regardless of women’s age. More importantly, these results provide a new perspective elucidating the mechanism of IVF successful rate by age, and motivate additional analyses to understand mechanisms that could describe the interplay between age and intermediary factors leading to live birth.

## Supplementary information

**Additional file 1** Sensitivity analyses, in order to assess the robustness of our estimates.

## Data Availability

The datasets used and/or analysed during the current study are available from the corresponding author on reasonable request.

## Abbreviations

AMH: anti-mullerian hormone
BMI: Body mass index
CI: Confidence interval
DE: Direct effect
E2: Estradiol
FSH: Follicle Stimulating Hormone
IE: indirect effect
IVF: In vitro fertilization
OR: Odds ratio
RR: Risk ratio
SD: Standard deviation
