# Supplementary material for: Age effect on in vitro fertilization pregnancy mediated by anti-Mullerian hormone (AMH) and modified by follicle stimulating hormone (FSH)
Source: BMC Pregnancy Childbirth. 2020 Apr 9;20:209. doi: 10.1186/s12884-020-02875-2 (PMC7147042; doi:10.1186/s12884-020-02875-2)
Supplement: Supplementary file 1 — Additional file 1 Sensitivity analyses, in order to assess the robustness of our estimates. [file 12884_2020_2875_MOESM1_ESM.pdf]

Supplementary Materials for "Age Effect on In Vitro Fertilization Pregnancy Mediated by Anti-Mullerian Hormone (AMH) and Modified by Follicle Stimulating Hormone (FSH)"

by

HC Hsieh, JY Su, S Wang and YT Huang

0.1 CONSORT diagram for data processing

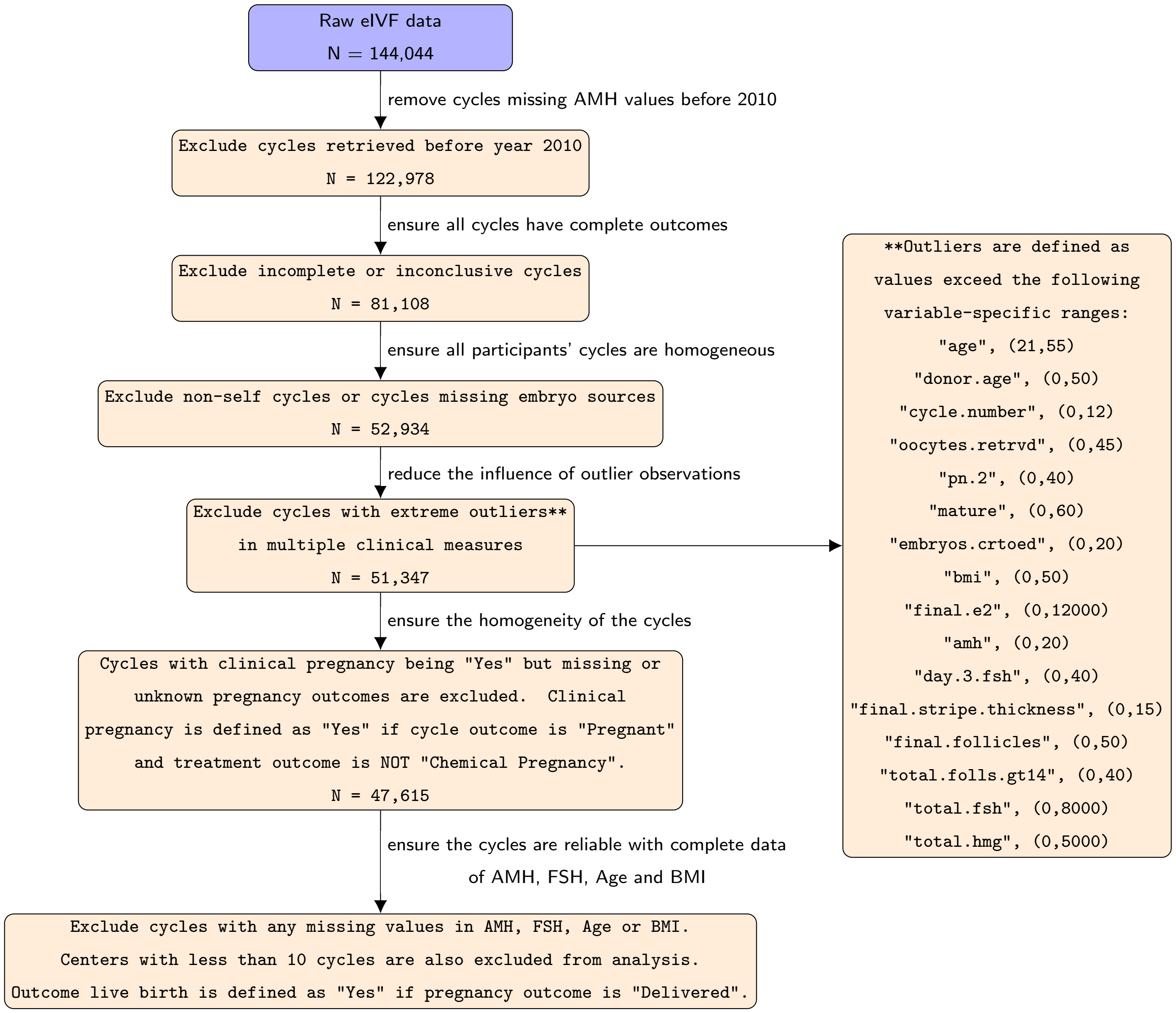

0.2 Sensitivity analysis

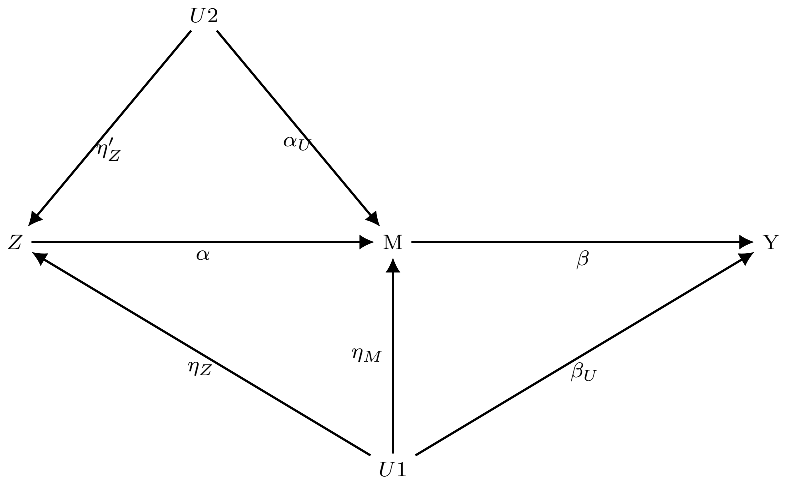

We conduct sensitivity analysis in order to assess the robustness of our estimates in the presence of potential violations against the assumptions and to quantify the magnitude of confounding for altering the observed results. Let U denote the confounder that should have been adjusted in the analyses but was not collected. We partition U into $\{U_1, U_2\}$ and propose models for $P(Y = 1|z_a, m, \boldsymbol{x}, U_1)$, $P(M = 1|z_b, \boldsymbol{x}, U_2)$ and confounders U_1, U_2:

$$\log\left(\frac{P(Y = 1|z_a, m, \boldsymbol{x}, U_1)}{1 - P(Y = 1|z_a, m, \boldsymbol{x}, U_1)}\right) = \boldsymbol{\beta}_X^T\boldsymbol{x} + \beta_M m + \beta_Z z_a + \beta_{ZM} z_a m + \beta_U U_1 \tag{1}$$

$$\log\left(\frac{P(M = 1|z_b, \boldsymbol{x}, U_2)}{1 - P(M = 1|z_b, \boldsymbol{x}, U_2)}\right) = \boldsymbol{\alpha}_X^T\boldsymbol{x} + \alpha_Z z_b + \alpha_U U_2 \tag{2}$$

where α_U is the log odds ratio of having M= 1 for one unit increase in U_2 and β_U is the log odds ratio of having Y = 1 for one unit increase in U_1, both are parameters for characterizing the magnitude of confounding bias. We assume the following models for the association between $\{U_1, U_2\}$ and $\{Z, M\}$:

$$U_1 = \eta_Z Z + \eta_M M + \epsilon_{U_1} \tag{3}$$

$$U_2 = \eta'_Z Z + \epsilon_{U_2} \tag{4}$$

Note that η_Z, η'_Z and η_M are parameters quantifying the magnitude of confounding where η_Z and η_M are the association of one unit increase in Z and M respectively with U_1; and η'_Z is the association of one unit increase in Z with U_2.
By approximating logistic distribution with normal distribution (Zeger et al., 1988), one can show that

$$\hat{P}(Y = 1|z_a, m, \boldsymbol{x}, U_1 = 0) = \text{expit}\left\{\log\left(\frac{\hat{P}(Y = 1|z_a, m, \boldsymbol{x})}{1 - \hat{P}(Y = 1|z_a, m, \boldsymbol{x})}\right)\sqrt{1 + 0.35 \times \beta_U^2} - \beta_U(\eta_Z z_a + \eta_M m)\right\} \tag{5}$$

$$\hat{P}(M = 1|z_b, \boldsymbol{x}, U_2 = 0) = \text{expit}\left\{\log\left(\frac{\hat{P}(M = 1|z_b, \boldsymbol{x})}{1 - \hat{P}(M = 1|z_b, \boldsymbol{x})}\right)\sqrt{1 + 0.35 \times \alpha_U^2} - \alpha_U(\eta'_Z z_b)\right\} \tag{6}$$

where expit(x)= $\frac{e^x}{1+e^x}$, $\boldsymbol{x}$ denotes BMI or other adjusted covariates, U_i denotes the confounder that should have been adjusted in the analyses but was not collected. With specification of $(\alpha_U, \beta_U, \eta_Z, \eta_M, \eta'_Z)$ and the estimates $\hat{P}(Y = 1|z_a, m, \boldsymbol{x})$ and $\hat{P}(M = 1|z_b, \boldsymbol{x})$ from the data, we can conduct sensitivity analyses using (5) and (6) to recover the unbiased estimates of direct and indirect effects. We design the following experiments to examine biases from various configurations of $(\alpha_U, \beta_U, \eta_Z, \eta_M, \eta'_Z)$:

1. Case 1: $-0.2 < \alpha_U < 0.2$, $-0.2 < \beta_U < 0.2$, and $\eta_Z = \eta_M = \eta'_Z = \eta$ where $\eta = -0.2$

2. Case 2: $-0.2 < \alpha_U < 0.2$, $-0.2 < \beta_U < 0.2$, and $\eta_Z = \eta_M = \eta'_Z = \eta$ where $\eta = 0.2$

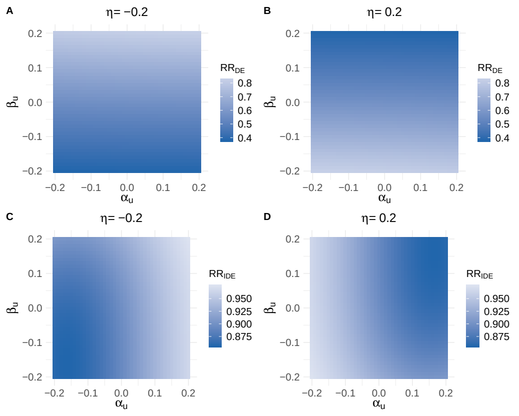

(a) AMH

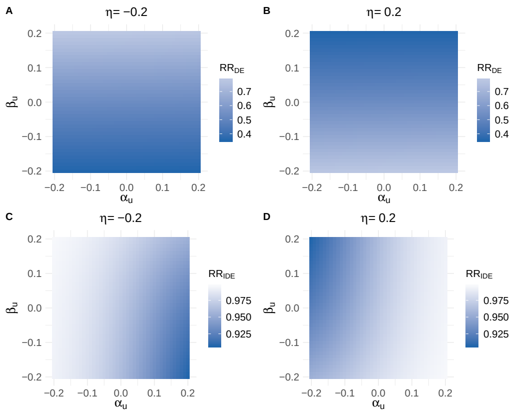

(b) FSH

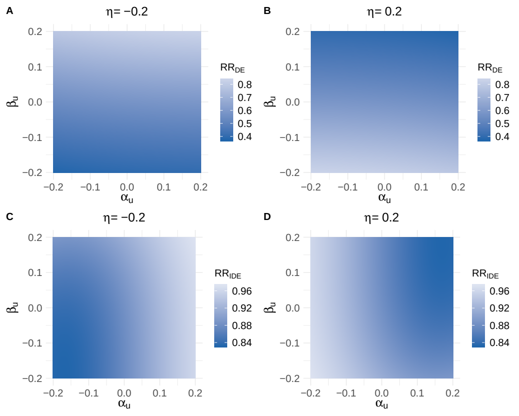

(c) AMH + AMH-by-age interaction

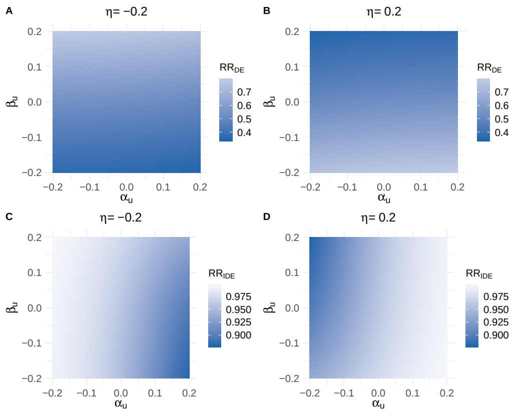

(d) FSH + FSH-by-age interaction

Figure 1: Sensitivity analysis for Table 4.

(a) and (c) are sensitivity analyses for direct and indirect effects through AMH; (b) and (d) are effects through FSH. Panels A and B are sensitivity analyses for direct effect, and panels C and D are for indirect effect.

Since age is recognized as the strongest known predictor of AMH, FSH and live birth rate, we fix the magnitude of the parameters according to the odds ratio of age for live birth.

In Case 1, the effects of U on the exposure and intermediate event were fixed to -0.2, and we found that the confounding effect had to be extremely large and negative, i.e., $\alpha_U = -0.2$ to nullify the observed indirect effect of age on live birth through FSH (Figure1.(b)C & 1.(d)C). On the other hand, we also found that the confounding effect had to be extremely large and positive, i.e., $\alpha_U = 0.2$ to nullify the observed indirect effect if we modify the level of FSH (Figure 2.(b)C). In Case 2, where the effects of U on the two events were fixed to 0.2, the results reveal that the confounding effect had to be extremely large and positive, i.e., $\alpha_U = 0.2$ to attenuate the indirect effect down to 1 (Figure1.(b)D & 1.(d)D). As for the indirect effect mediated by AMH, even the association of the confounder with the intermediate event and the outcome event is as large as -0.2 or 0.2, the observed indirect effect is still less than 1 (Figures 1.(a)C&D and 1.(c)C&D). For the indirect effect through AMH stratified by FSH, the observed effect under FSH < 9.7 may decrease to $RR = 1$ if both η and α_U have absolute value of 0.2 and are in opposite directions (Figures 2.(a)-(d), panels C &D)

In summary, the sensitivity analyses suggest that the effect may shrink to zero only if effects of the confounder on both events and the association of the confounder with AMH/FSH and live birth are extremely large, which is unlikely to occur. Therefore, we conclude that our estimates deriving from mediation analyses of age on live birth through AMH or FSH are robust.

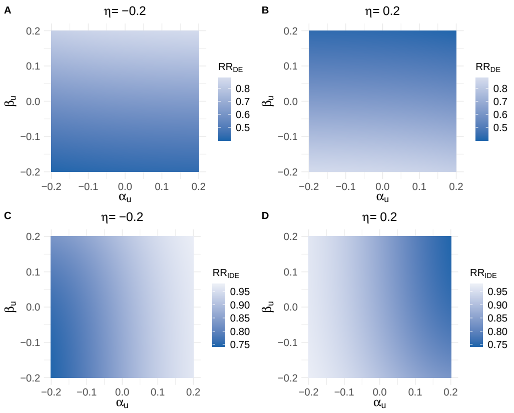

(a) FSH < 5.1

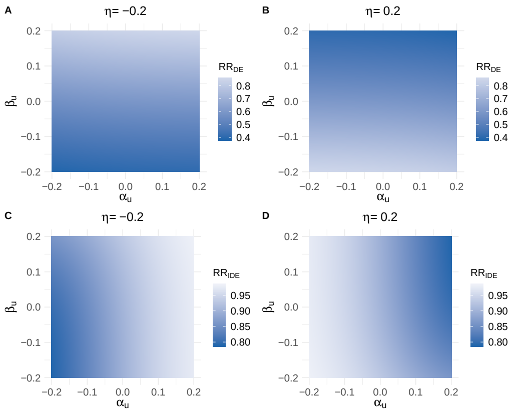

(b) 5.1 <FSH< 6.5

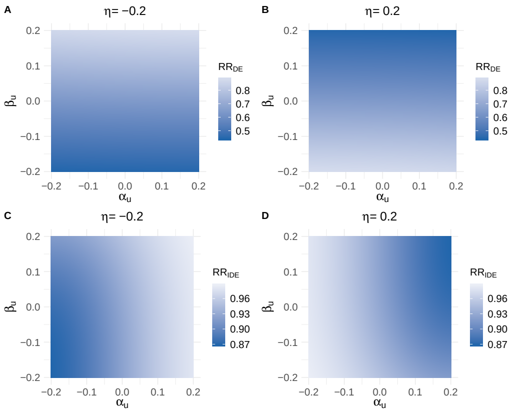

(c) 6.5 <FSH< 7.7

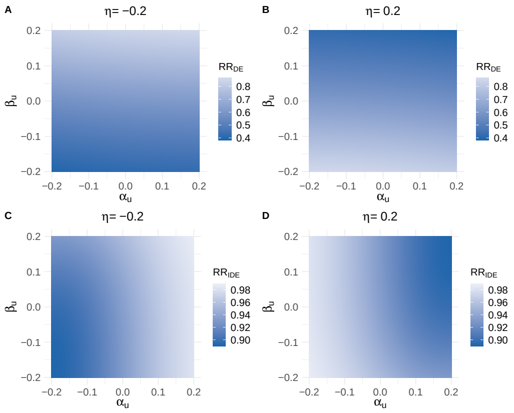

(d) 7.7 <FSH< 9.7

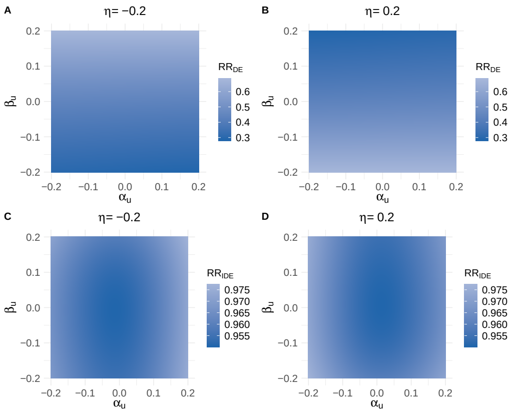

(e) FSH> 9.7

Figure 2: Sensitivity analysis for Table 5.

(a)-(e) are under different FSH levels. Panels A and B are sensitivity analyses for direct effect and panels C and D are for indirect effect.
